# Azo-Bridged Dextran: A Photoresponsive Sustainable Material with Photo-Tunable Mechanical Properties

**DOI:** 10.1021/acs.biomac.4c01508

**Published:** 2025-02-06

**Authors:** Konstantin Knaipp, Rupert Kargl, Damjan Makuc, Janez Plavec, Ema Žagar, Karin Stana Kleinschek, Georg Gescheidt

**Affiliations:** † Institute of Physical and Theoretical Chemistry, TU Graz, Stremayrgase 9, A-8010 Graz, Austria; ‡ Institute of Chemistry and Technology of Biobased Systems, TU Graz, Stremayrgase 9, A-8010 Graz, Austria; § Slovenian NMR Centre, National Institute of Chemistry, Hajdrihova 19, Sl-1000 Ljubljana, Slovenia; ∥ Faculty of Chemistry and Chemical Technology, University of Ljubljana, Večna Pot 113, SI-1000 Ljubljana, Slovenia; ⊥ EN-FIST Center of Excellence, Trg Osvobodilne Fronte 13, SI-1000 Ljubljana, Slovenia; # Department of Polymer Chemistry and Technology, National Institute of Chemistry, Hajdrihova 19, Sl-1000 Ljubljana, Slovenia; △ Institute of Automatisation, Faculty of Electrical Engineering and Computer Science, University of Maribor, Koroška cesta 46, SI-2000 Maribor, Slovenia

## Abstract

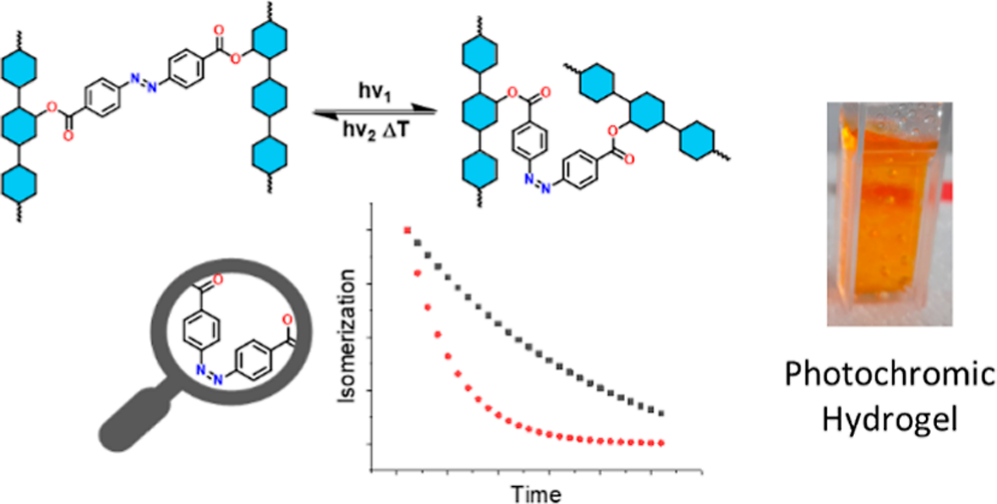

We report on the synthesis, characterization, and properties of dextran polymers, which are covalently bridged/cross-linked by azobenzene moieties. The reversible photoactivity of the azo moiety is retained in the polymers, and the kinetics of the *E*/*Z* isomerization depend on the dextran/azobenzene ratio. Together with the simple preparation, our approach provides convenient access to photoresponsive sustainable materials. Moreover, based on the water-soluble polymers, we have prepared photoresponsive hydrogels, which soften upon UV irradiation. Our findings are based on spectroscopy (UV/vis, IR, and NMR/DOSY), size exclusion chromatography, and rheology.

## 1 Introduction

Photoresponsive biomaterials provide a broad range of applications.^1−4^ Natural polysaccharides are well suited in this field based on their nontoxicity, high biocompatibility, and biodegradability.^5^ Accordingly, over the last years, many polysaccharide-based materials, in particular hydrogels, for use in drug delivery,^6^ tissue engineering,^7^ and wound healing^8^ have been reported. Suitable polymer networks are a decisive starting point for developing smart materials, and understanding their stimulus-response is crucial.^9,10^ A polysaccharide with a long history of use in biomedicine is dextran. Its good water solubility, nontoxicity, and low antigenicity were first exploited in its most classic biomedical application, which is the use of aqueous dextran solutions as a replacement for blood plasma.^11^ In its cross-linked form, dextran is most often encountered as the chromatographic medium Sephadex. More recent research has focused on employing dextran-derived materials for drug delivery,^12^ tissue engineering scaffolds,^13^ wound dressings,^14^ and biosensing applications.^15^

Here, we show the access to photoresponsive molecular networks expanding the scope of dextran-based materials.^16^ We use covalent cross-linking with azobenzene (4,4-azobenzene dicarboxylic acid; **B**), a well-established molecular photoswitch. This allows selectively creating *E* or *Z* isomers of the covalently embedded azobenzene moieties^17^ using distinct wavelengths. It is established that the presence of either an *E* or *Z* azobenzene component determines the characteristics of the materials. This has been demonstrated, e.g., in the fields of self-healing polymers^18^ and sensing,^19^ for the photocontrol of enzyme conformation,^20^ for energy storage applications,^21^ and for responsive surfaces or (bio)polymers.^22^ The aim of this work is to establish whether the photochromic character of azobenzene is retained in dextran networks and if it leads to distinguishable photochromic properties of the new materials.

To this end, we present the synthesis of azobenzene-bridged dextran and explore the photoresponse of the polymers. Moreover, we examined the possibility of producing dextran/azobenzene hydrogels.

## 2 Synthetic Strategy

**Scheme 1:**
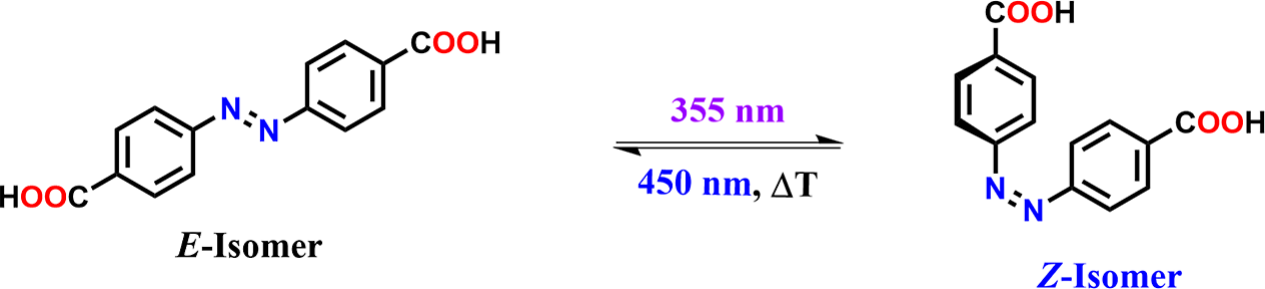
*E*/*Z* Photoisomerization of the Bifunctional 4,4-Azobenzene Dicarboxylic Acid (**B**)

For the synthesis of the cross-linked dextran polymers, we use azobenzene-4,4′-dicarboxylic acid (**B**). The *E*-isomer of **B** possesses absorption maxima at wavelengths of 440 and 320 nm. Its energy is 10–12 kcal/mol lower than that of the corresponding *Z* isomer. Correspondingly, a solution of **B** that is kept in the dark will consist almost entirely of the *E*-isomer. When a solution of the *E*-isomer is illuminated at 355 nm, the *E* to *Z* photoisomerization takes place. This is associated with a change in the UV/vis spectrum of the solution, since the *Z* isomer has absorption maxima at 430, 280, and 250 nm. Upon light irradiation the rates of *E* to *Z* and *Z* to *E* isomerization eventually equilibrate, a photostationary state (PSS) is reached, containing an excess of the *Z* isomer. The reverse process can be triggered by illumination at a wavelength of 450 nm, eventually establishing an *E*-enriched PSS (see Scheme 1, and UV/vis spectra are shown in Figure 5). The differences between the two isomers are not limited to their UV/vis spectra, with the *Z* isomer possessing a bent geometry, in which the phenyl rings are twisted out-of-plane with the azo group.^23^

For decades, azobenzene dyes have been used as colorants in the textile and food industries,^24^ and owing to their photoactivity, much research has been conducted on bioconjugates of these molecules. The conjugation of azobenzene to polysaccharides can be accomplished by the formation of amides,^25^ esters, ethers, or carbamates.^26^ Moreover, glycosidic bonds with saccharides can be formed starting from *p*-aminophenol reacting to azobenzene derivatives.^27^ One possibility to form an ester linkage between 4,4-azobenzene dicarboxylic acid (**B**) and the hydroxyl groups of the polysaccharide is the activation of the carboxylic acid using 1,1′-carbonyldiimidazole **(CDI)**, a technique that can be applied to a wide range of carboxyl containing compounds such as *N*-protected amino acids.^28,29^

Dextran-based nanogels have been prepared by grafting monofunctional azobenzene moieties onto the dextran backbone, making use of the hydrophobic interaction between the grafts.^30^ It has further been reported that physical cross-linking between cyclodextrin-modified dextran and a diazobenzene linker can be accomplished using host–guest interactions.^31^ A 4-arm polyethylene glycol and 4,4-azobenzene dicarboxylic acid were used to cross-link cellulose nanofibers, resulting in a photochromic smart hydrogel that could release bovine serum albumin when the *E* to *Z* photoisomerization was induced via UV light.^32^ It is apparent that the reaction of 4,4-azobenzene dicarboxylic acid with dextran will result in the formation of an unprecedented photoresponsive network whose synthesis and properties are elucidated in the present work.

## 3 Experimental Section

### 3.1 Synthesis

#### 3.1.1 General Procedure for Obtaining Azobenzene-Cross-linked Dextran

4,4′-Azobenzene dicarboxylic acid (**B**) was dissolved in DMSO (20 mL). Two equivalents (relative to **B**) of **CDI** dissolved in DMSO (5 mL) were added dropwise until gas evolution stopped. The mixture was added to a solution of dextran (**D**, *M*_w_ 20 kDa, in 25 mL of DMSO) and stirred at elevated temperature. The product was precipitated by the addition of ethanol (100 mL); the precipitate was filtered off and washed with EtOH (100 mL). After drying overnight over solid KOH, the product was obtained as an eggshell-colored powder.

The amounts of the reactants and reaction conditions are listed in the Supporting Information (Table S1). Analogous reactions were performed with monofunctional 4-azobenzene carboxylic acid (**M**) using one equivalent of **CDI** for control (see Table S1). The corresponding products are denominated as **DBD** (weight portion of the linker) and **DM** for the cross-linked and the reference derivatives, respectively.

#### 3.1.2 General Procedure for the Synthesis of the Gels

Linker **B** and dextran (**D**, *M*_w_ 20 kDa) were suspended in DMSO and stirred for 2 h at 80 °C. A solution of **CDI** in DMSO was added dropwise. The mixture was stirred until all particles had dissolved, and the evolution of gas had ceased. The amounts of the reactants and reaction conditions are presented in the Supporting Information (Table S2). The solution was stored at 80 °C for 19 h. The product was immersed into deionized H_2_O. The water was exchanged 5 times over the course of 3 days, and the swollen hydrogels were stored underwater.

#### 3.1.3 UV/Vis Spectroscopy and Illumination

UV/vis spectra were recorded on a fiber-optic spectrophotometer (J&M Analytik AG, Esslingen, Germany, UV/vis spectrometer equipped with a 1024-pixel diode array detector, light sources: deuterium and halogen lamps). The soluble composites were dissolved in deionized H_2_O, and spectra were recorded in quartz cuvettes (*d* = 1 cm). **B** and **M** were dissolved in pH = 9.2 borate buffer. Gels were filled into disposable polystyrene cuvettes (*d* = 0.3 cm) or squeezed tightly between two microscope slides depending on the optical density of the material. The used illumination setup was described previously.^33^ By placing an LED perpendicular to the light path of the spectrophotometer, it allows simultaneous illumination of the sample and recording of UV/vis spectra. A detailed description of the LEDs, including emission spectra and measured light fluxes, is available in the Supporting Information. The gels were illuminated with a UV-lamp (Lightningcure LC4, Hamamatsu Photonics, Japan).

#### 3.1.4 Elemental Analysis

The C/N/H/S content of the lyophilized gels was determined using a Vario MICRO cube elemental analyzer (Elementar Analysensysteme GmbH, Langenselbold, Germany). Two mg samples were analyzed in duplicate. Sulfanilamide was used as a calibration standard, along with He as a carrier and oxygen as the combustion gas.

#### 3.1.5 Determination of **B**/**M** Content

The cross-linker content of the soluble composites was determined via photometry by comparing the absorbance at 365 nm to that of a solution of **B** or **M**. The **B** content of the gels was determined from elemental analysis under the assumption that all nitrogen was contained within the azo groups of **B**.

#### 3.1.6 IR Spectroscopy

IR spectra were recorded on a FT-IR spectrometer (Bruker ALPHA, Billerica, USA) operated in ATR mode. The composites were investigated after drying, and the gels were lyophilized before recording the spectra.

#### 3.1.7 ^1^H NMR and ^1^H DOSY

^1^H DOSY spectra were recorded on a 600 MHz Avance NEO spectrometer (Bruker BioSpin GmbH, Ettlingen, Germany) using the standard Bruker *stebpesgp1s* pulse sequence. Sixteen different gradient strengths were incremented between 1 and 47 G/cm for the DOSY gradient array with a gradient pulse duration of 4.4 ms. The diffusion delay between coding and decoding gradient pulse was optimized and set to 250 ms. Each gradient increment was recorded using 96 scans. The spectra were processed using Dynamics Center 2.8.0.1 software.

^1^H DOSY spectra for the determination of diffusion constants were recorded on a 400 MHz Avance III 400 NMR spectrometer (Bruker BioSpin GmbH, Ettlingen, Germany) using the *stegp1s* pulse sequence. Samples were dissolved in D_2_O (10 mg/mL). Spectra were integrated from 3.995 to 3.28 ppm and fitted with the TopSpin software suite.

#### 3.1.8 Size Exclusion Chromatography

The molar mass characteristics of samples were determined by size-exclusion chromatography coupled with a multiangle light scattering detector (DAWN HELEOS-II, Wyatt Technology Corporation, USA) and a refractive index detector (Optilab T-rEX, Wyatt Technology Corporation, USA) (SEC/MALS-RI). The input parameter required to determine the molar mass characteristics of HPMC is a specific refractive index increment (*dn*/*dc*), which was determined assuming 100% mass recovery of the samples from the column. Separation of samples by size was performed at room temperature on an Agilent 1260 HPLC chromatograph (Agilent Technologies, USA) using a PolarGel-L analytical column (7.5 mm × 300 mm, 8 μm) with a precolumn (both columns from Agilent Laboratories, USA) and aqueous 0.1 M NaNO_3_ solution with pH = 10 as a mobile phase. The nominal flow rate of the eluent was 1 mL min^–1^. The sample solutions with a concentration of ∼1 mg mL^–1^ were injected directly onto the SEC column, so that the mass of the injected sample was ∼100 μg. Astra 7.3.1 software (Wyatt Technology Corp., USA) was used for data acquisition and evaluation.

#### 3.1.9 Rheological Measurements

All rheological measurements were performed in a rheometer using a plate–plate setup (Anton Paar Group AG, Graz, Austria). The plate diameter was 25 mm, and the plate–plate distance was 0.5 mm. The sample temperature was maintained at 25 °C. Amplitude tests were conducted from 0.01 to 100% shear deformation at an angular frequency of 10 rad/s. Frequency tests were conducted within a range of 0.1 to 100 rad/s. The shear deformation was 1%, within the LVE region of all of the investigated gels.

## 4 Results and Discussion

### 4.1 Synthesis and Evaluation

**Scheme 2:**
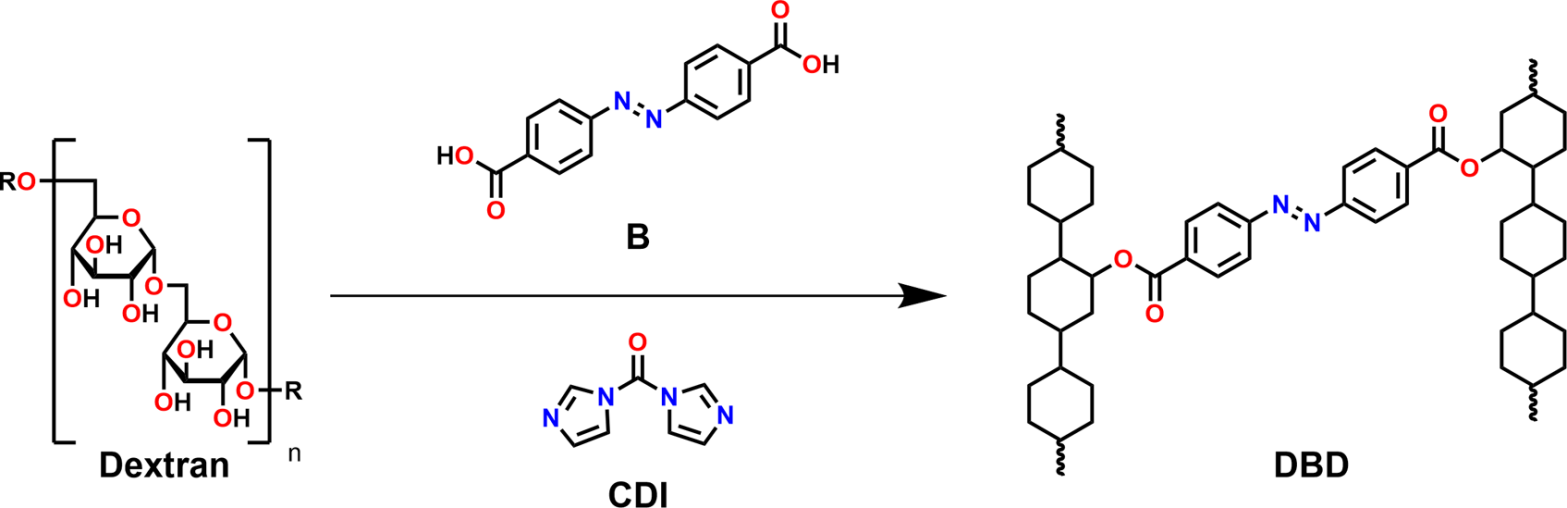
Coupling Reaction of **B** to Dextran

In 2008, Wondraczek and Heinze^29^ showed that attaching a carboxylic acid to dextran (**D**) can be conveniently accomplished by using **CDI** as the coupling agent. Following this procedure, we have used 4,4′-azobenzene dicarboxylic acid (**B**) to perform links between dextran moieties (Scheme 2).

We initially performed the reaction in a dilute DMSO solution with various **B**/**D** ratios. After precipitation with EtOH, we obtained various water-soluble cross-linked products, which are denoted **DBD** with the mass percentage of **B** in brackets. Yields and degrees of substitution for all products are listed in the Supporting Information. The highest degree of cross-linking obtained in this way was **DBD**(7.4), as higher contents of **B** led to insoluble products. As a control, the reaction was also performed with 4-azobenzene carboxylic acid (**M**), yielding the **DM** product.

At higher educt concentrations, the reaction mixtures solidified into DMSO-containing solvogels. These were turned into hydrogels by exchanging the solvent over several days. In this way, we obtained the less-cross-linked **DBD**(9.9) and the highly cross-linked **DBD**(16.6) gels.

**Figure 1:**
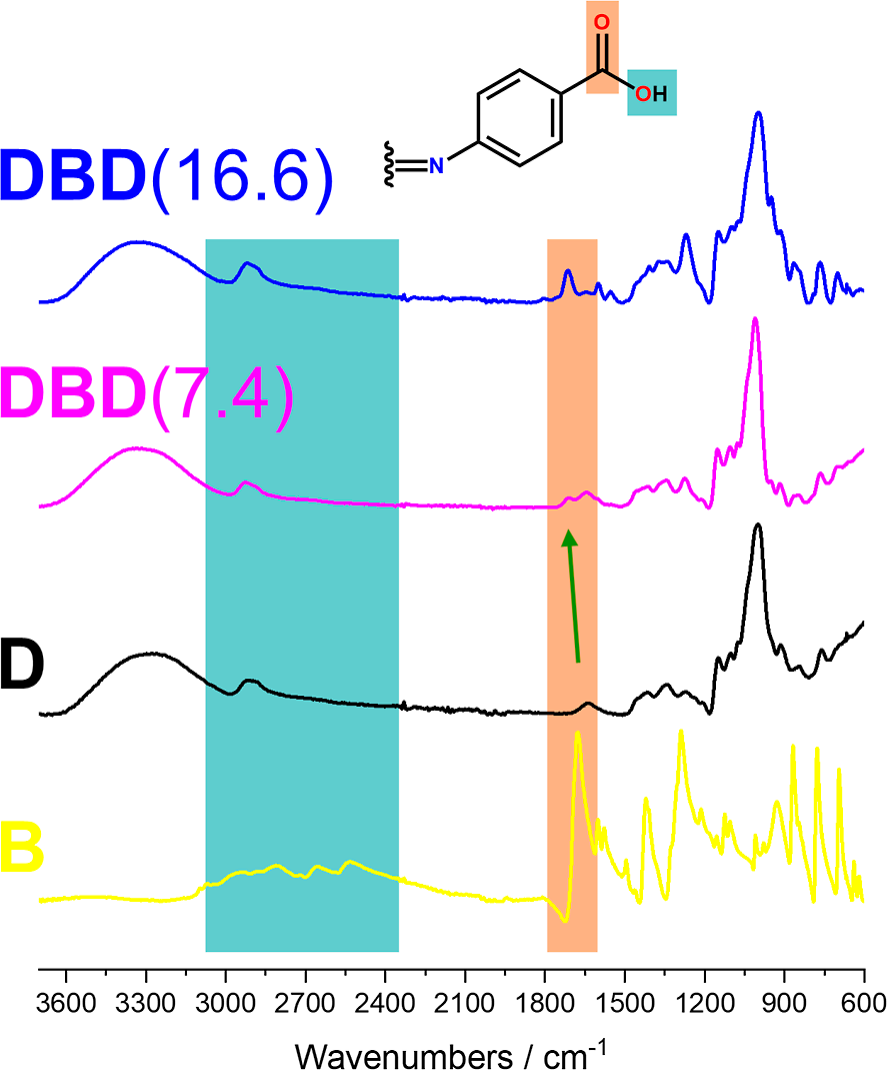
FTIR spectra of **B**, **D**, **DBD**(7.4), and **DBD**(16.6).

To establish the mode of binding between **D** and **B**, we have performed FT-IR, diffusion NMR, and SEC analyses. The IR spectra of unmodified dextran, **D**, linker **B**, the cross-linked product **DBD**(7.4), and the lyophilized gel **DBD**(16.6) are shown in Figure 1. The IR spectrum of **D** is dominated by the O–H stretching modes (from 3600 to 3000 cm^–1^), skeletal CH_2_ stretching vibrations (at 2900 cm^–1^), and C–O deformations (around 1000 cm^–1^). Due to the high molecular weight and the abundance of hydrogen bonds, the vibrational modes of dextran are highly broadened.^34^ The cross-linker **B** possesses two modes that are characteristic for carboxylic acids. The C=O stretching mode at 1677 cm^–1^, while the O–H stretching modes are broadened, reaching from 3100 to 2500 cm^–1^.^35^

Owing to the low cross-linker content in the **DBD** derivatives, the vibrational modes of **B** are only clearly distinguishable in the IR spectra of highly cross-linked derivatives like **DBD**(7.4) and **DBD**(16.6). The disappearance of the O–H mode of **B** suggests that a dominating portion of the carboxyl OH group was coupled to **D**. This is further substantiated by the C=O stretching mode, which shifts from 1677 to 1715 cm^–1^, being characteristic of esters of carboxylic acids.

A possible side reaction is the cross-linking of dextran through the formation of carbonate linkages.^36,37^ However, the absence of C=O stretching modes at higher wavenumbers (1740 to 1760 cm^–1^),^35^ being characteristic for alkyl carbonates, indicates that this side-reaction only plays a minor role.

**Figure 2:**
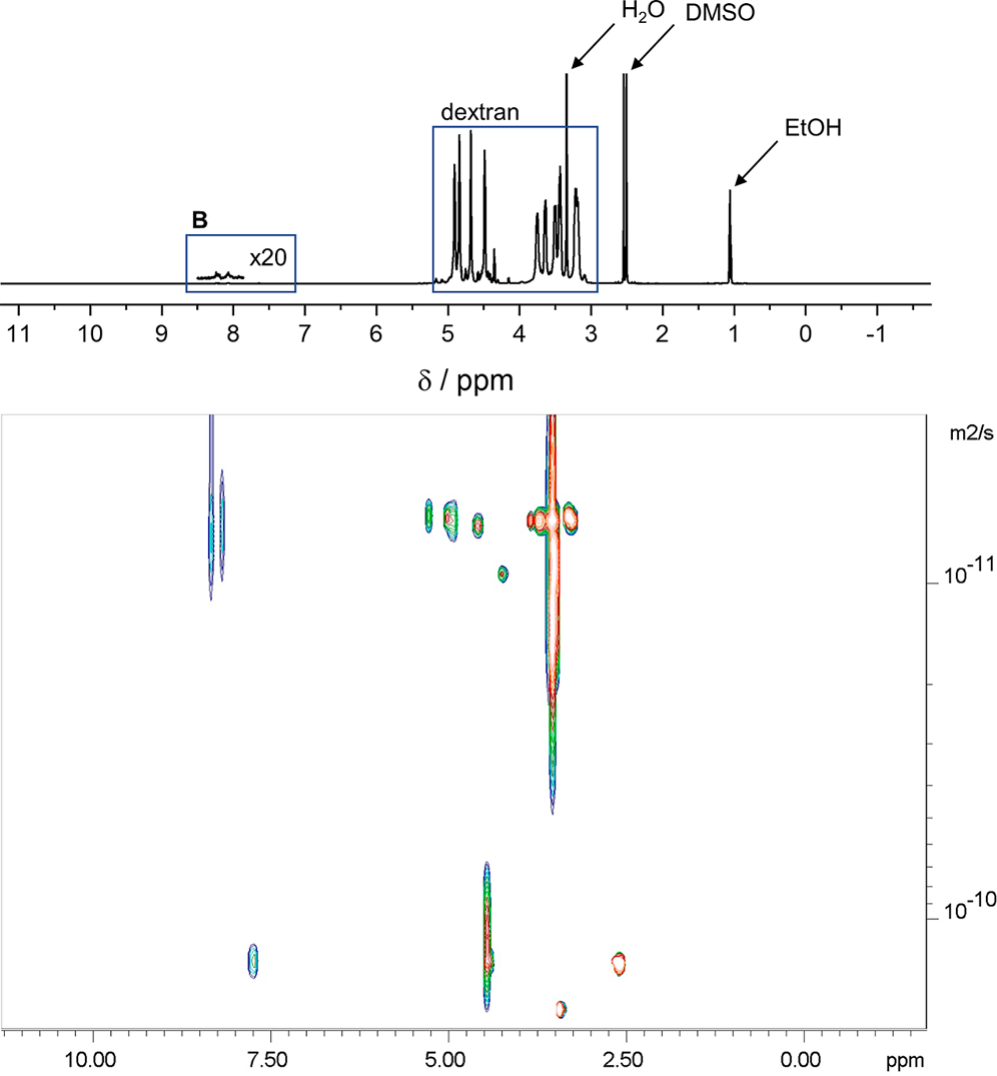
^1^H NMR (top) and ^1^H DOSY (bottom) spectrum of **DBD**(0.3) in DMSO-*d*_6_.

The ^1^H NMR spectrum of **DBD**(0.3) in DMSO-*d*_6_ is shown in Figure 2. The ^1^H NMR spectrum of dextran is characterized by a cluster of peaks ranging from 3.2 to 3.8 ppm, which can be attributed to the non-anomeric protons bound directly to the skeletal carbons (C2 to C6). The peak of the anomeric proton is shifted downfield due to the α-glycosidic linkage. It appears together with the protons of the hydroxyl moieties (OH2, OH3, and OH4), which are visible due to the absence of exchange broadening in DMSO-*d*_6_.^38^ The NMR spectrum of cross-linker **B** reveals two doublets in the aromatic region (*J* = 8.5 Hz) at 8.01 and 8.17 ppm. The ^1^H NMR spectrum of **DBD**(0.3) basically reflects the sum of the two components (with the appropriate intensity ratios).^39^

The NMR-DOSY experiment can separate NMR peaks according to their associated diffusion constant. The observation (see Figure 2) that resonances assigned to the cross-linker indicate the same diffusion constant as those of the skeletal protons of the dextran moiety provides strong evidence for covalent attachment of **B** to **D**.

IR and NMR spectroscopy has shown that the –COOH groups of **B** were esterified with the –OH groups of dextran. However, it is unclear how much of this conjugation happens within one dextran molecule (intramolecular cross-linking). To establish the degree of intramolecular cross-linking, we have correlated the diffusion constants, *D*, determined by DOSY, with the corresponding molecular weights determined via SEC.

**Figure 3:**
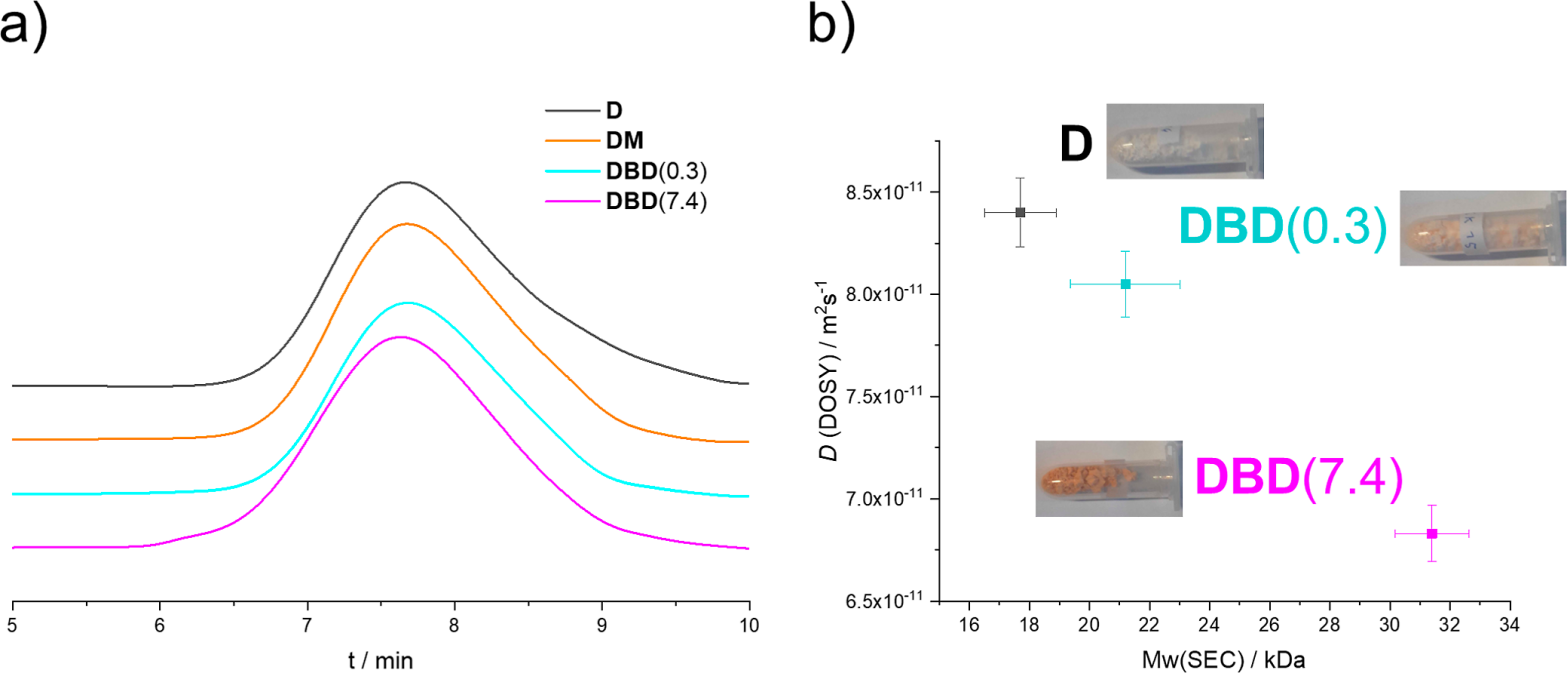
(a) SEC of native dextran **D**, a lightly cross-linked derivative **DBD**(0.3), a highly cross-linked derivative **DBD**(7.4), and of the non-cross-linked sample **DM**. Apart from the chromatogram for **DBD**(7.4), which is shifted toward smaller elution times, the obtained curves are mostly identical. (b) Plot of diffusion constants determined by DOSY NMR against the *M*_w_ obtained via SEC, showing the linear relationship between the two properties. Photographs indicate the corresponding samples.

The SEC chromatograms are shown in Figure 3. The molecular weight of our parent dextran samples was determined by SEC to be 17 kDa. Upon functionalization with the monoacid **M**, no changes in the *M*_w_ are detected. Whereas at small **B**/**D** ratios, the SEC-determined molecular weight remains basically unchanged; it clearly increases for **DBD**(7.4), yielding 31.4 kDa (Table 1). This is clearly visible in the chromatogram of **DBD**(7.4), which shows a shift toward smaller elution times.

**Table 1:** Comparison of Molecular Weights (as Determined by SEC), Diffusion Constants, and Hydrodynamic Radius (as Determined by NMR-DOSY) for the Different Composites

	*M*_w_/kDa	*D*/10^–11^ m^2^s^–1^	*r*_s_/10^–9^ m
dextran	17.7 ± 1.2	8.4 ± 0.17	2.09 ± 0.04
**DBD**(0.3)	21.2 ± 1.8	8.05 ± 0.16	2.18 ± 0.04
**DBD**(7.4)	31.4 ± 1.2	6.8 ± 0.14	2.58 ± 0.05

These results correlate well with the values of the diffusion constant and hydrodynamic radius obtained from NMR-DOSY experiments (Table 1). According to the Stokes–Einstein relationship (eq 1), the diffusion constant *D* is inversely proportional to the hydrodynamic radius *r*_s_. Since *r*_s_ increases with *M*_w_, *D* is intimately connected to the molecular weights obtained via SEC. The other parameters in the Stokes–Einstein relationship are the Boltzmann constant *k*, the absolute temperature *T*, and the viscosity of the solvent η. The plot shown in Figure 3 indicates proportionality between *M*_w_ and *D* in line with previously reported data for polysaccharides^40^
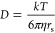
1

The rather similar molecular weights determined for **D** and **DBD**(0.3) (17.7 and 21.7 kDa, respectively) point to a substantial amount of intramolecular cross-linking in **DBD**(0.3). The higher *M*_w_ of **DBD**(7.4) indicates that the number of intermolecular cross-links increases with a higher content of **B**. This finding is substantiated by the corresponding hydrodynamic volumes (i.e., *D* values, Table 1 and Figure 3). No drop in the *M*_w_ of the products is observed in the SEC measurements, which indicates no degradation under the experimental conditions.

### 4.2 Hydrogels

We observed that higher educt concentrations led to DMSO-containing solvogels. Following solvent exchange with water, we obtained the less cross-linked **DBD**(9.9) and the highly cross-linked **DBD**(16.6) hydrogels.

IR spectroscopy (see Figure 1) gives no indication that apart from their higher content of **B**, the hydrogels **DBD**(9.9) and **DBD**(16.6) are chemically distinct from the soluble derivatives. The different morphology of the gels arises from the higher educt concentration during synthesis and can be investigated by probing the mechanical properties of the materials in a rheological experiment.

**Figure 4:**
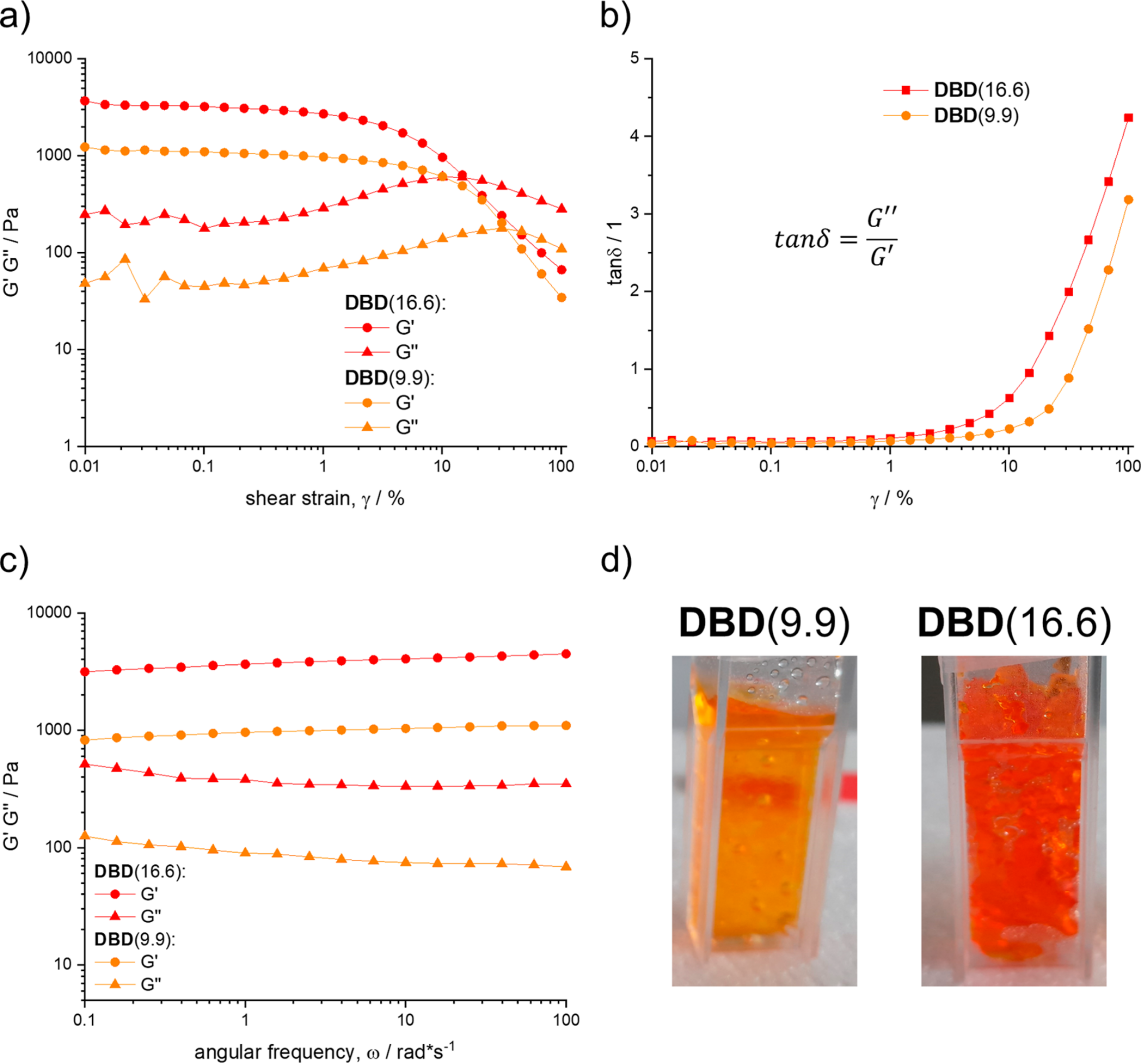
Amplitude scan (a), loss tangent (b), frequency scan (c), and photographs (d) of **DBD**(9.9) and **DBD**(16.6). Error bars omitted for clarity (see Supporting Information).

It is apparent that differences in cross-linker content directly affect the mechanical strength of the gels. **DBD**(16.6) is stiff and breaks apart when force is applied to it, while **DBD**(9.9) is more flexible. These differences can be quantified by determining the rheological properties of the hydrogels.^41^Figure 4 shows the amplitude scans and loss tangents (tan δ) of both **DBD**(9.9) and **DBD**(16.6).

Both **DBD** derivatives show typical behavior for cross-linked hydrogels (Figure 4). At low shear rates, both gels behave as solids (*G*′ > *G*″). As the shear rate increases, the gels gradually start to soften until the curves for *G*′ and *G*″ crossover, and the gel behaves as a liquid (tan δ > 1). For both gels, the linear-viscoelastic region (LVE) stretches to shear rates of approximately 2%. Due to its lower degree of cross-linking, **DBD**(9.9) possesses smaller *G*′ and *G*″, it is a softer, more flexible material than **DBD**(16.6). The distinct “hump” in the *G*″ curve is characteristic for hydrogels that possess a continuous superstructure. As the shear rate begins to increase, microfissures start to form in the material. The freely moving elements around the fissures are capable of dissipating large amounts of energy, while the overall structure of the gel remains intact. This leads to the observed rise in *G*″. At the maximum value of *G*″, a macrofissure forms in the material, and the gel begins to flow like a liquid. The frequency scans of the gels (Figure 4c) show that *G*′ remains above *G*″ within the entire measured frequency range. The absence of a crossover point between the two curves is characteristic for a covalently cross-linked hydrogel. As shown above, the stiffer **DBD**(16.6) possesses higher values of *G*′ and *G*″ than the more flexible **DBD**(9.9) gel.

The rheological properties of the gels point toward the existence of a covalently cross-linked superstructure within the materials. This suggests that the amount of intermolecular cross-linking between dextran molecules is much higher than in the soluble products synthesized in dilute solution.

### 4.3 Optical Properties and Photo-Induced Activity

**Figure 5:**
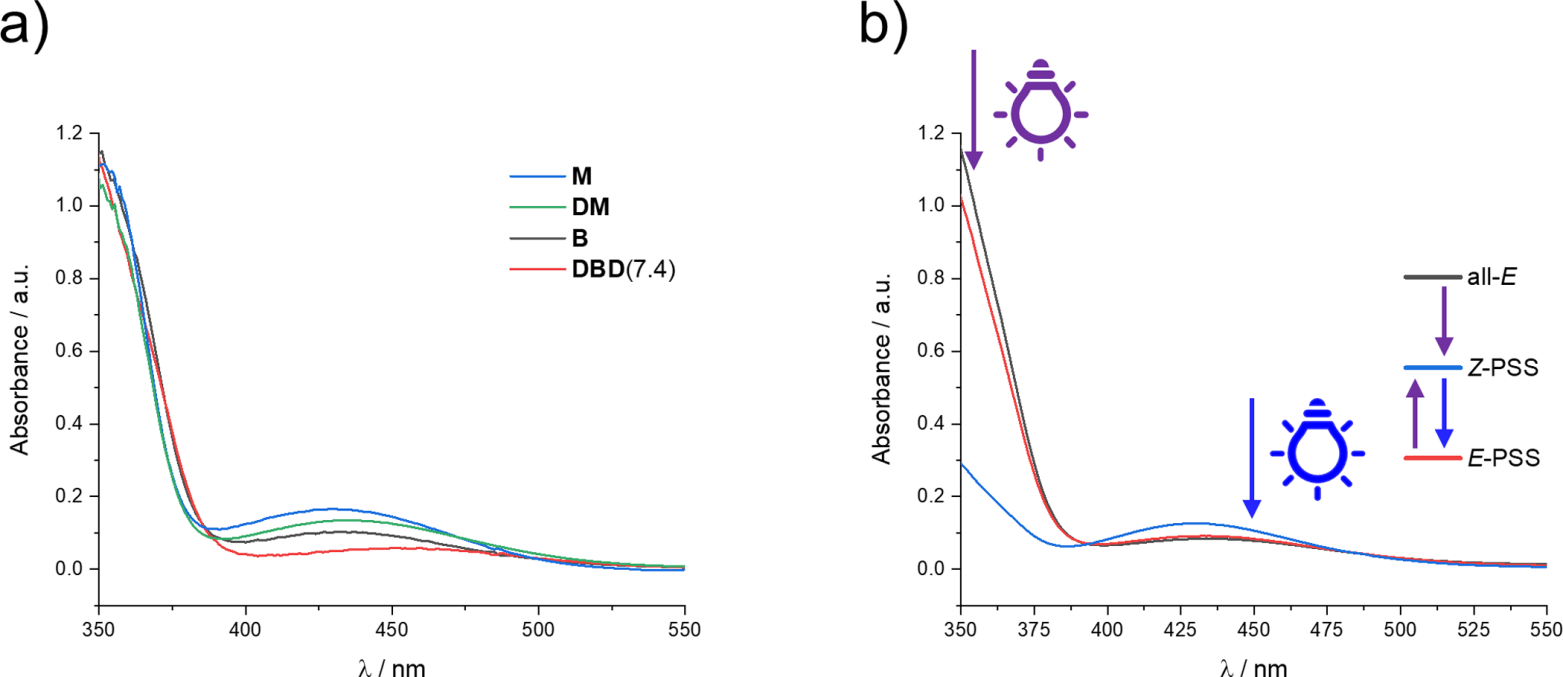
(a) UV–vis spectra of **B** and **M** (aqueous pH = 9 borate buffer), as well as **DBD**(7.4) and **DM** (recorded in water). (b) In black: spectrum of a freshly prepared solution of **B** (aqueous pH = 9 borate buffer). In blue: spectrum after irradiation at 355 nm. In red: spectrum after subsequent irradiation at 450 nm.

A comparison of the UV–vis spectra of **M**, **DM**, **B**, and **DBD**(7.4) is shown in Figure 5a. While the position of the π–π* band does not change upon esterification with dextran, the n–π* band decreases in intensity and shifts toward slightly higher wavelengths. This effect is more pronounced for **B** (430 to 460 nm) than for **M** (430 to 435 nm).

Upon illumination, solutions of **M**, **B**, **DM**, and of all soluble **DBD** derivatives display similar photoswitching behavior. Irradiation at 355 nm leads to the conversion of the parent *E* isomer toward *Z* until an equilibrium, the PSS is reached. As a representative example, Figure 5b indicates the changes in the UV–vis spectra of **B** recorded upon illumination at 355 nm, followed by irradiation at 450 nm. Analogous behavior is displayed by **M**, **DM**, and the different **DBD** derivatives. Upon irradiation at 355 nm, the absorbance of the π–π* band decreases, while the n–π* band increases in intensity and shifts slightly toward lower wavelengths. When this latter solution is irradiated at 450 nm, a PSS is reached, which is dominated by the *E* isomer.

**Figure 6:**
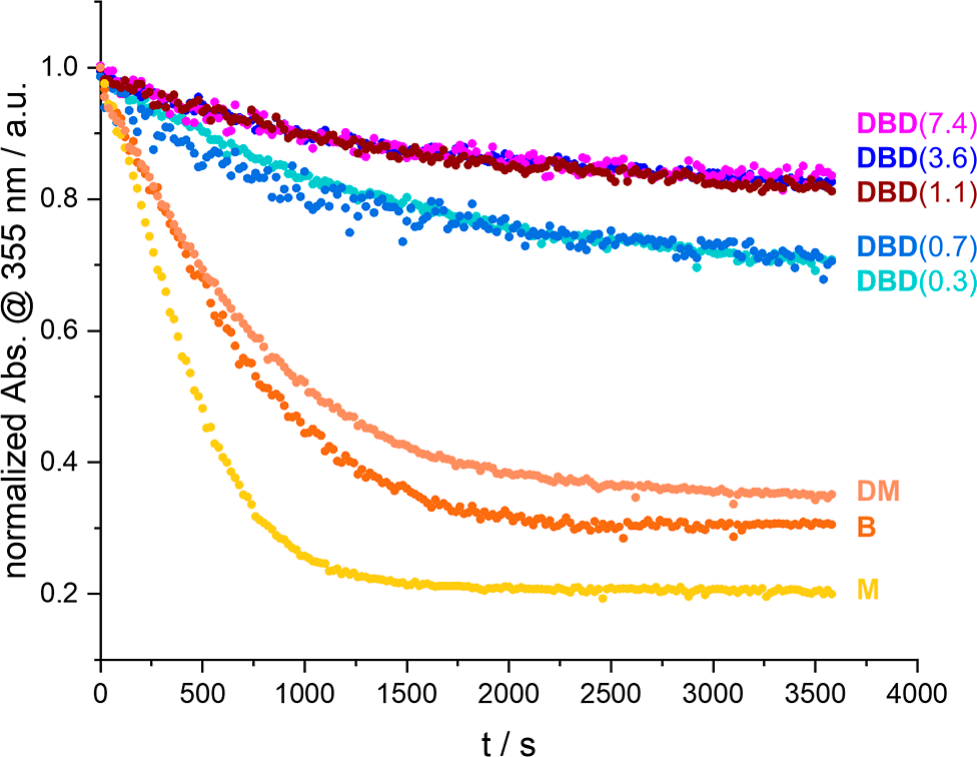
Time traces at 355 nm for the *E* to *Z* conversion of **DBD** samples compared to the references **DM**, **B**, and **M**.

To evaluate the photoswitching ability of the dextran-bound azobenzene moieties, we have recorded time profiles for parent compounds **M** and **B**, dextran coupled to a monoacid (**DM**) and cross-linked derivatives (**DBD**) with various linker/dextran ratios. Figure 6 shows the normalized absorbance at 355 nm over the course of the illumination for the *E* to *Z* conversion of freshly prepared (all *E*) solutions. It is apparent that each sample eventually reaches a PSS, but that the kinetics of the photoswitching process differ.

**Scheme 3:**
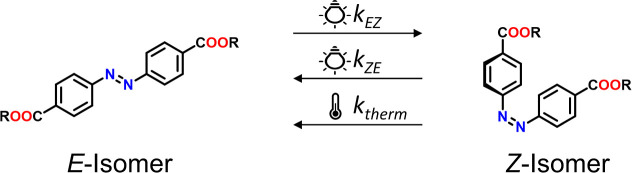
Reactions That Occur Upon Illumination of an Azobenzene Solution: Photochemical *E* to *Z* and *Z* to *E* Conversion As Well As Thermal *Z* to *E* Conversion

The photoswitching kinetics of azobenzenes result from three simultaneous reactions.^42^ When a solution of azobenzenes is illuminated, the overlap in the absorption spectra leads to simultaneous absorption of light by both isomers. Accordingly, the *E* to *Z* and *Z* to *E* photoisomerizations always occur together. Furthermore, there is a constant thermal reaction converting the *Z* isomer into the more energetically favorable *E* isomer (Scheme 3).

The three reactions can be regarded as first-order reactions. Here, the first one describes the *E* to *Z* conversion (see eq 2). The change in the concentration of the *E* isomer (*c*_*E*_) depends on the light absorbed by the *E* isomer (*I*_*E*_), the rate constant for *E* to *Z* photoisomerization (*k*_*EZ*_), and the concentration of the *E* isomer. Consequently, the rate of the *Z* to *E* photoisomerization (see eq 3) depends on the concentration of the Z isomer (*c*_*Z*_), the light absorbed by the *Z* isomer (*I*_*Z*_), and the associated rate constant (*k*_*ZE*_). The thermal *Z* to *E* isomerization solely depends on the rate constant *k*_therm_ (see eq 4)
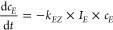
2
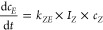
3
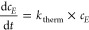
4

When the solution is continuously illuminated, the system reaches a dynamic equilibrium called a PSS. Since *k*_*EZ*_, *k*_*ZE*_, *I*_*E*_, and *I*_*Z*_ depend on the wavelength of the incident light, different illumination wavelengths may produce PSSs with different isomer ratios.

UV–vis spectra of *Z*-PSS solutions taken several hours after illumination show no change compared with spectra recorded immediately after illumination. Therefore, the thermal *Z* to *E* conversion is not rate determining while the solution is illuminated. A freshly prepared solution contains no *Z* isomer. Thus, the E isomer produces an absorption at 355 nm. Accordingly, the absorption at 355 nm is directly proportional to *c*_*E*_, and the rate law can be simplified to the following expression (eq 5), where *I* is the overall light flux and *A*_355_ is the absorbance at 355 nm
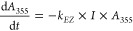
5

**Figure 7:**
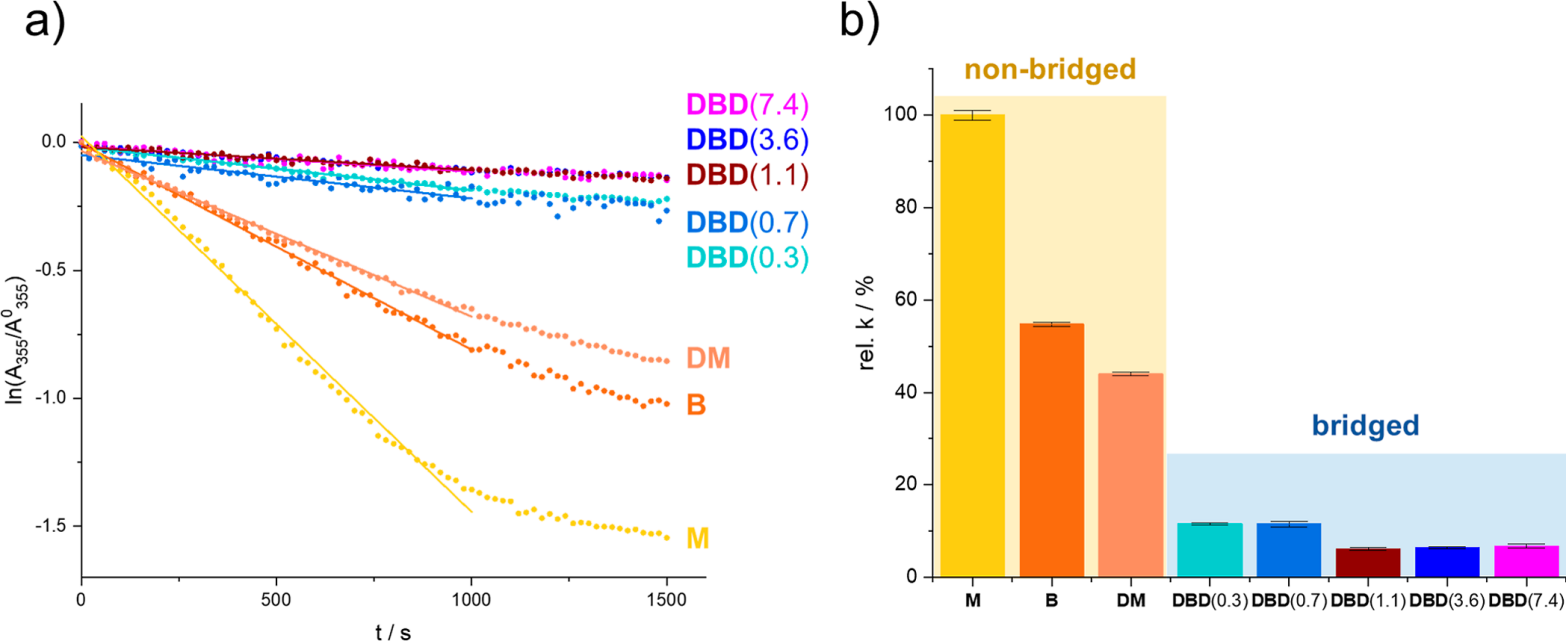
(a) Logarithmic plot of the curves shown in Figure 6 (the regions used for the fit are marked with the corresponding lines). (b) Relative rate constants for reference compounds **M**, **B**, **DM**, and **DBD**(0.3–7.4); error bars are taken from the linear fits.

This is illustrated in Figure 7a, which shows the curves displayed in Figure 6 on a logarithmic scale. At lower conversions, the kinetics clearly follow first order, as expected for the *E* to *Z* isomerization of azobenzene. Owing to the growing contribution of the *Z* isomer to A_355_, the linear behavior of the plot gradually deteriorates. Accordingly, we used decays only up to 1000 s to determine the corresponding first-order rate constants. They are compared in Figure 7b. For simplicity (see the Supporting Information for the absolute values), we have set the value of parent **M** as the reference (100%). For dicarboxylic acid **B**, the rate constant is 55% relative to that of **M** and that of **DM** is only slightly lower (44%). Substantially lower rate constants follow for the dextran derivatives, which are reacted with the bifunctional **B**. Whereas the rate constant for **DBD**(0.3/0.7) reaches 12% of the reference it is further reduced to 6% for **DBD**(1.1/3.6/7.4).

The considerations shown above only apply to the *E* to *Z* conversion; analyzing the *Z* to *E* isomerization in the same way yields similar results (see Supporting Information). The observed drop in *k*_*EZ*_ resembles similar observations made in azobenzene-containing amphiphilic compounds, where *k*_*EZ*_ dropped when the critical micelle concentration was surpassed.^43^ In the case of the amphiphilic azobenzenes, this was rationalized as the effect of two azobenzene populations, one with high *k*_*EZ*_ outside the micelles and one with low *k*_*EZ*_ contained in micelles. Contrary to the amphiphilic azobenzenes, in this work, the *k*_*ZE*_ of the **DBD** derivatives was unaffected by the concentration. This suggests a different mechanisms in the **DBD** derivatives. We observed that all **DBD** derivatives undergo slower photoswitching than free **B** and that the overall difference in absorbance between the two PSS is smaller than in free **B**. Therefore, we assume that large portions of the **B** cross-linker do not participate in photoswitching and instead serve as an internal filter that attenuates the light. This may be caused by an increase in the local rigidity in the polymer network, even at only partial *E* to *Z* conversion of the azo moieties.

It is remarkable that photoswitching activity is observed here, as azobenzenes serving as structural elements inside highly ordered reticular frameworks often exhibit no photoswitching ability,^44^ continuously revert to the *E* isomer due to induced stress within the framework,^45^ or undergo a ligand-buckling transition instead.^46^ It is likely that the observed photoswitching behavior is only possible due to the soft and amorphous nature of dextran together with the relatively low amount of cross-linking.

It must be kept in mind that *k*_*EZ*_ is a compound parameter that also includes the absorption cross-section (what proportion of incident light is absorbed) and the quantum yield (what proportion of absorbed photons leads to isomerization). Additionally, for photoreactions, the overall rates also depend on the light intensity. Accordingly, by adjusting the light intensity *I*, the isomerization rate can be conveniently adjusted.

**Figure 8:**
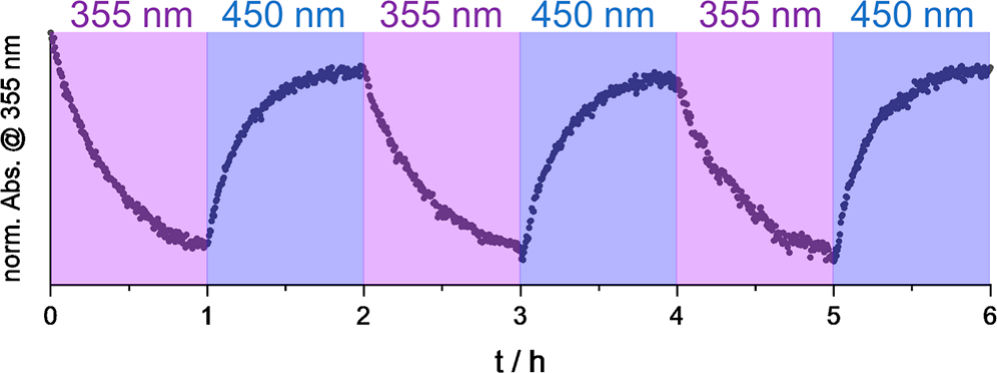
Cyclic irradiation of **DBD**(0.7) at 355 nm followed by 450 nm at pH = 7.

Figure 8 shows the photo response of **DBD**(0.7) toward irradiation cycling at 355/450 nm. We observe that the sample can be cycled repeatedly for 6 h, within this time frame, no bleaching of the chromophore or change in the photoswitching kinetics is observed. This points toward a high degree of stability in the **B**-**D** linkage, with little degradation at neutral pH. When the experiment is conducted in an aqueous borate buffer (pH = 9.2), the photoswitching behavior of **DBD**(0.7) gradually approaches that of free **B**, as the ester bonds are hydrolyzed (see Supporting Information).

The photoswitching experiments conducted with the soluble composites reveal that a high content of **B** is associated with a low *k*_*EZ*_. Furthermore, the gels cannot be diluted, meaning that high light fluxes are required for the isomerization. Therefore, the chosen light source was a high-power UV lamp, which illuminated the sample from various angels for 30 min. To prevent photothermal heating, the sample holder was cooled with compressed air. While **DBD**(9.9) could be measured in a polystyrene cuvette, the high optical density of **DBD**(16.6) required the material to be squeezed between two microscope slides to resolve the n–π* band.

**Figure 9:**
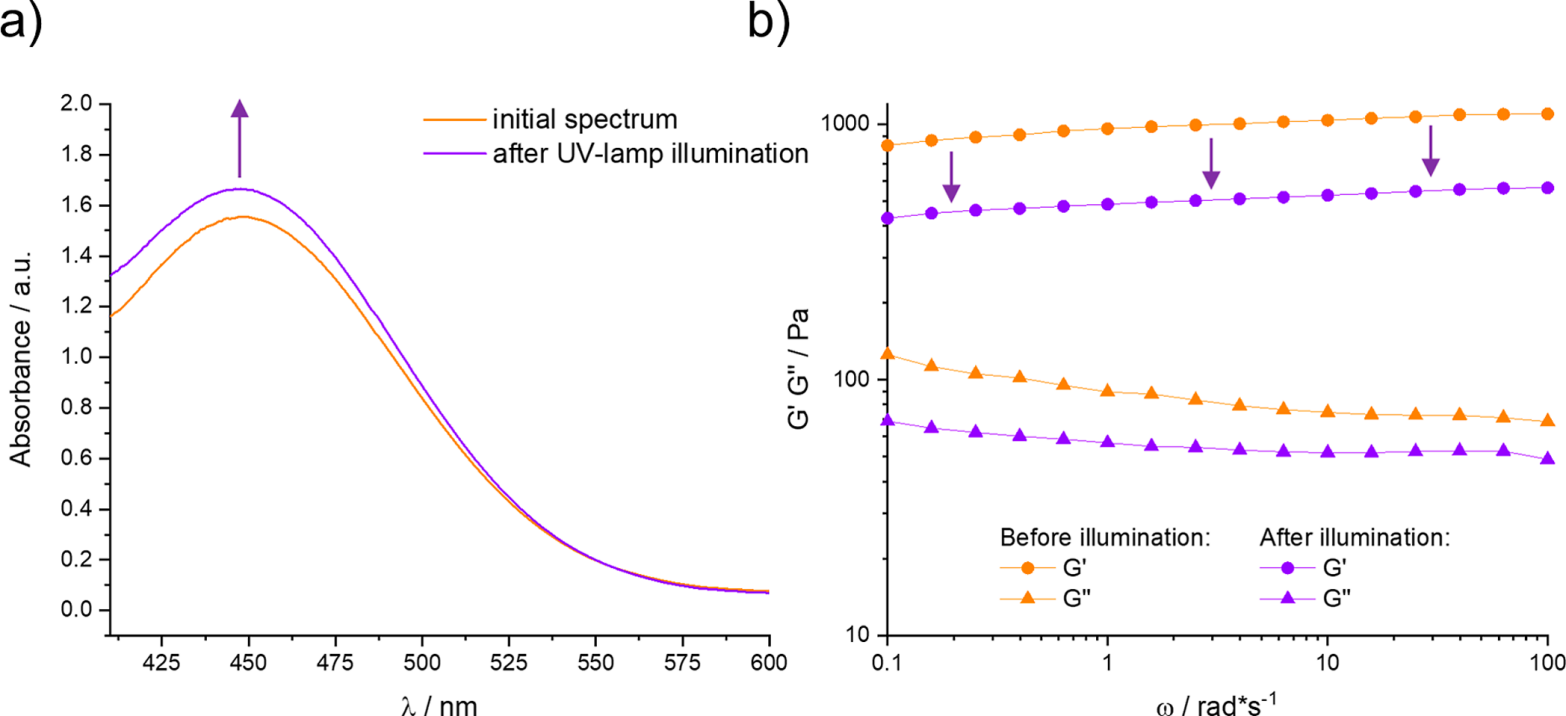
(a) UV/vis spectrum before (orange) and after (violet) illumination with a UV lamp for 30 min. (b) Photo-dependent frequency scans of the **DBD**(9.9) gel before (orange) and after (violet) illumination.

We observe that the *E* to *Z* photoisomerization is also possible in gels **DBD**(9.9) and more rigid gel **DBD**(16.6). Figure 9 displays frequency scans taken before and after UV irradiation for **DBD**(9.9). *G*′ and *G*″ drop (by approximately 40%) when the sample is enriched with the *Z* isomer. This indicates that the *E* to *Z* photoisomerization of the covalently bound azo moiety causes a well-detectable softening of the hydrogel. This being in line with previously published observations of acrylate-based hydrogels cross-linked by azobenzenes.^47^ The observed drop in *G*′ and *G*″ is much smaller in **DBD**(16.6) (just 15%, see Supporting Information). Studies on similar systems have shown that the change in mechanical properties upon illumination depends on the azobenzene cross-linker content.^48^ Not only are the mechanical properties of the synthesized gels tunable, but this tunability also extends to the mechanical photoresponse.

## 5 Summary

Dextran can be conveniently cross-linked with the bifunctional azo linker **B**. The azo-bridged, water-soluble dextran polymers retain the photoactivity of the azo group. The rates of *E* to *Z* isomerization depend on the ratio between the linker and the polymer and decrease to ca. 10–5% compared with the free linker B. The time scale of the isomerization can be adjusted by the intensity of the LEDs. The *E* to *Z* procedure is fully reversible. Accordingly, azo-bridged dextran of the type **DBD** have the potential to be used as components in photoactive materials.

The use of highly concentrated solutions yields gels. We converted the primary DMSO-based gel to a hydrogel. Even in such hydrogels, the photoactivity of the azo group is retained. In this latter case, *E* to *Z* isomerization of the azo moieties causes a change of the mechanical properties: The *E*-enriched material softens upon its conversion to the *Z*-based material related to the properties of self-healing azo-based polymers.

Our investigations illustrate that dextran-based photoresponsive materials can be conveniently synthesized, and accordingly, this opens a route to new sustainable functional devices with a plethora of applications.

## Supporting Information Available

Detailed synthesis information; calculated yields and degree of substitution values for all products; LEDs, including their spectral profile and measured light fluxes; absolute values for *k*_*EZ*_, as well as a kinetic evaluation of the *Z* to *E* photoisomerization; and individual rheological measurements for the two hydrogels, including error bars and the photoresponse of the **DBD**(16.6) gel (PDF)
