# Supplementary material for: Azo-Bridged Dextran: A Photoresponsive Sustainable Material with Photo-Tunable Mechanical Properties
Source: Biomacromolecules. 2025 Feb 6;26(3):1737–47. doi: 10.1021/acs.biomac.4c01508 (PMC11898052; doi:10.1021/acs.biomac.4c01508)
Supplement: Supplementary file 1 — bm4c01508_si_001.pdf [file bm4c01508_si_001.pdf]

Azo-Bridged Dextran: A Photoresponsive Sustainable Material with Photo-Tunable Mechanical Properties

Supporting Information

Konstantin Knaipp[a], Rupert Kargl[b], Damjan Makuc[c], Janez Plavec[c,d,e], Ema Žagar[f], Karin Stana Kleinschek[b,g], Georg Gescheidt[a]**

a) Institute of Physical and Theoretical Chemistry, TU Graz, Stremayrgase 9, A-8010 Graz, Austria

b) Institute of Chemistry and Technology of Biobased Systems, TU Graz, Stremayrgase 9, A-8010 Graz, Austria

c) Slovenian NMR Centre, National Institute of Chemistry, Hajdrihova 19, SI-1000 Ljubljana, Slovenia

d) Faculty of Chemistry and Chemical Technology, University of Ljubljana, Večna pot 113, SI-1000 Ljubljana, Slovenia

e) EN-FIST Center of Excellence, Trg Osvobodilne fronte 13, SI-1000 Ljubljana, Slovenia

f) Department of Polymer Chemistry and Technology, National Institute of Chemistry, Hajdrihova 19, SI-1000 Ljubljana, Slovenia

g) Institute of Automatisation, Faculty of Electrical Engineering and Computer Science, University of Maribor, Koroška cesta 46, SI-2000 Maribor, Slovenia

* Email:
g.gescheidt-demner@tugraz.at
karin.stanakleinschek@tugraz.at

Contents

1. Synthesis details 3
1. Degree of substitution and yield 4
2. LEDs 5
3. Absolute values of k_{EZ} 6
4. Analysis of *Z* to *E* switching 7
5. Supplementary hydrogel information 9
6. Cyclic irradiation of **DBD**(0.7) in alkaline buffer 11
7. Photoresponse of **DBD**(16.6) 12

1. Synthesis details

The following tables contain details about the synthesis conditions of the dextran-azobenzene products. Table S1 shows the experimental conditions for the synthesis of the soluble derivatives, while Table S2 shows the conditions for the synthesis of the gels.

Table S1. Synthesis conditions for the soluble composites.

Name of product	m(**D**)	m(**B**/**M**)	m(CDI)	Temperature	Duration
**DBD**(0.3)	506 mg	52.1 mg	61.8 mg	60°C	6 h
**DBD**(0.7)	503 mg	82.2 mg	98.0 mg	60°C	6 h
**DBD**(1.1)	511 mg	108 mg	132 mg	60°C	6 h
**DBD**(3.6)	259 mg	258 mg	308 mg	60°C	6 h
**DBD**(7.4)	518 mg	101 mg	124 mg	80°C	18 h
**DM**	504 mg	101 mg	73.0 mg	60°C	6 h

Table S2. Synthesis conditions for the gels

	m(**D**)	m(**B**)	m(CDI)	V(**D**+**B**)	V(CDI)
**DBD**(9.9)	2120 mg	299 mg	354 mg	10 mL	2 mL
**DBD**(16.6)	2022 mg	421 mg	511 mg	7 mL	2 mL

2. Degree of substitution and yield

In polymer chemistry the degree of functionalisation (DS) describes the ratio of grafted substituents to monomer units. In this case the ratio of **B** and **M** to the anhydrous glucose units (AGUs). Since **B** can bind to two AGUs it is counted twice. It is assumed that all carboxylic groups of **B** and **M** are esterified.

Table S3. Calculated degrees of substitution for the different products.

	DS / %
**DBD**(0.3)	0.4
**DBD**(0.7)	1.0
**DBD**(1.1)	1.5
**DBD**(3.6)	5.1
**DBD**(7.4)	11
**DM**	0.3
**DBD**(9.9)	15
**DBD**(16.6)	27

Table S4. Obtained yields of the soluble **DBD** composites. The relative yields are obtained by comparing the amount of recovered product to the amount of utilized educt.

	Absolute yield / mg	Rel. yield dextran / %	Rel. yield **B**/**M** / %
**DBD**(0.3)	439	87	2.5
**DBD**(0.7)	297	59	2.5
**DBD**(1.1)	479	94	4.9
**DBD**(3.6)	195	75	2.7
**DBD**(7.4)	451	87	33
**DM**	444	88	1.3

3. LEDs

To induce *E*->*Z* isomerization the samples were illuminated with a 355 nm LED (XSL-355-5E-R6, Roithner Lasertechnik, Vienna) operated at 40 mA. This resulted in a light flux of 2.34*10^{-6} molL^{-1}s^{-1}. *Z*->*E* isomerization was induced with a 450 nm LED (LED450-05 Roithner Lasertechnik, Vienna), when operated at 0.5 mA (low-power setting) the light flux was 6.35*10^{-7} molL-1s-1, when operated at 10 mA (high-power setting) the light flux was 8.72*10^{-6} mols^{-1}L^{-1}. All light flux measurements were performed with a spectroradiometer (GL Spectis, GLOptics).

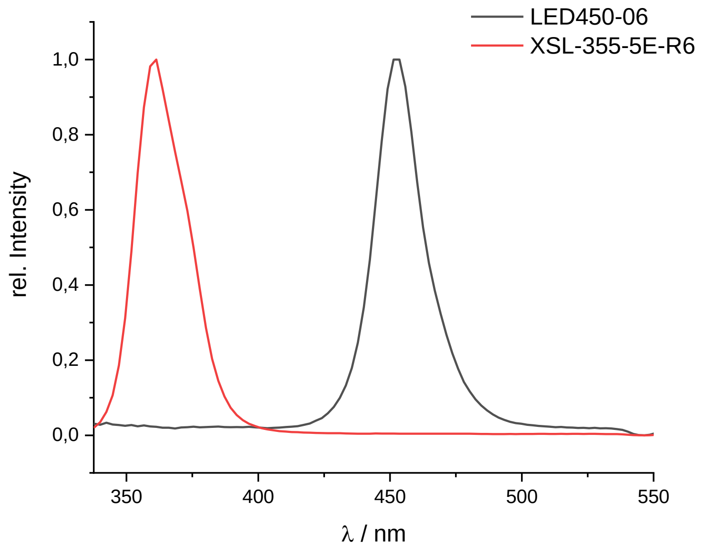

Figure S1. Normalized emission profiles of the used LEDs.

4. Absolute values of k_{EZ}

Table S5. Absolute values for the slope of the fitting curves of the *E* to *Z* isomerization (k_{EZ}*I) and the values corrected for the LED light flux. Errors taken from linear fits.

	k_{EZ}*I / s^{-1}	k_{EZ} / mol*s^{-2}*l^{-1}
**M**	$1.47*10^{-3} \pm 16*10^{-6}$	628 ± 7
**B**	$0.805*10^{-3} \pm 7*10^{-6}$	344 ± 3
**DM**	$0.647*10^{-3} \pm 5*10^{-6}$	276 ± 2
**DBD**(0.3)	$0.170*10^{-3} \pm 3*10^{-6}$	72.6 ± 1.3
**DBD**(0.7)	$0.169*10^{-3} \pm 9*10^{-6}$	72 ± 4
**DBD**(1.1)	$0.0901*10^{-3} \pm 4*10^{-6}$	39 ± 2
**DBD**(3.6)	$0.0938*10^{-3} \pm 3*10^{-6}$	40 ± 1.4
**DBD**(7.4)	$0.0989*10^{-3} \pm 6*10^{-6}$	42 ± 3

5. Analysis of *Z* to *E* switching

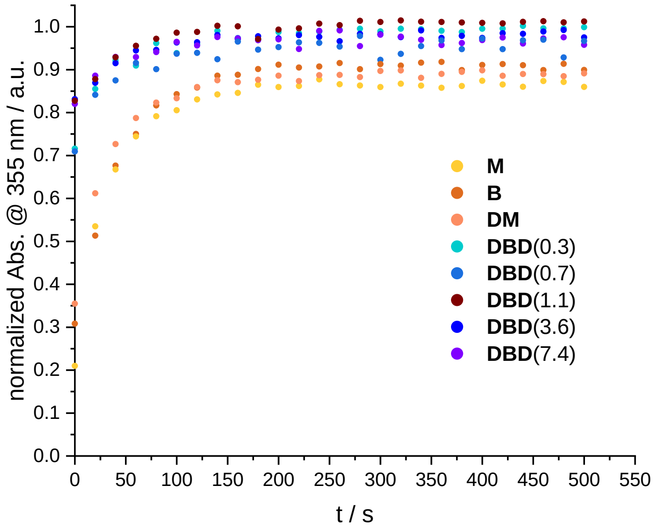

Figure S2. Time traces at 355 nm for the *Z* to *E* conversion of the investigated samples. Illumination at 450 nm, high-power setting.

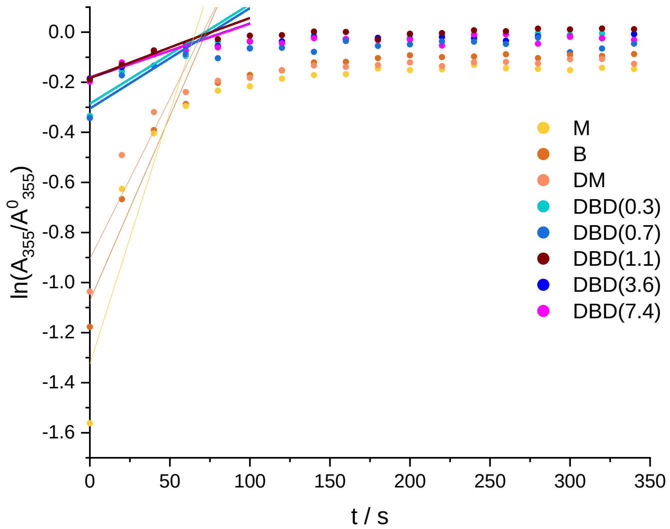

Figure S3. Logarithmic plot of the curves shown in Figure 2 (the regions used for the fit are marked with the corresponding lines).

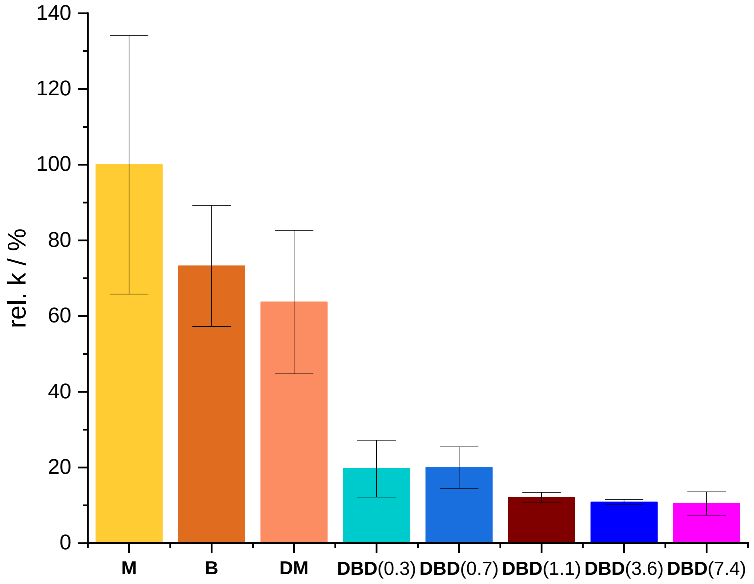

Figure S4. Relative rate constants for reference compounds **M**, **B**, and **DM**, and **DBD**(0.3–7.4); error bars are taken from the linear fits.

Table S6. Absolute values for the slope of the fitting curves of the *Z* to *E* isomerization (k_{EZ}*I) and the values corrected for the LED light flux. Errors taken from linear fits.

	k_{ZE}*I / s^{-1}	k_{ZE} / mol*s^{-2}*l^{-1}
**M**	20*10^{-3} ± 7*10^{-3}	2.3*10^{3} ± 0.8*10^{3}
**B**	15*10^{-3} ± 3*10^{-3}	1.7*10^{3} ± 0.4*10^{3}
**DM**	13*10^{-3} ± 4*10^{-3}	1.5*10^{3} ± 0.4*10^{3}
**DBD**(0.3)	4*10^{-3} ± 1.5*10^{-3}	0.5*10^{3} ± 0.2*10^{3}
**DBD**(0.7)	4*10^{-3} ± 1*10^{-3}	0.5*10^{3} ± 0.1*10^{3}
**DBD**(1.1)	2.4*10^{-3} ± 0.3*10^{-3}	0.28*10^{3} ± 0.02*10^{3}
**DBD**(3.6)	2.2*10^{-3} ± 0.1*10^{-3}	0.25*10^{3} ± 0.016*10^{3}
**DBD**(7.4)	2.1*10^{-3} ± 0.6*10^{-3}	0.24*10^{3} ± 0.07*10^{3}

6. Supplementary hydrogel information

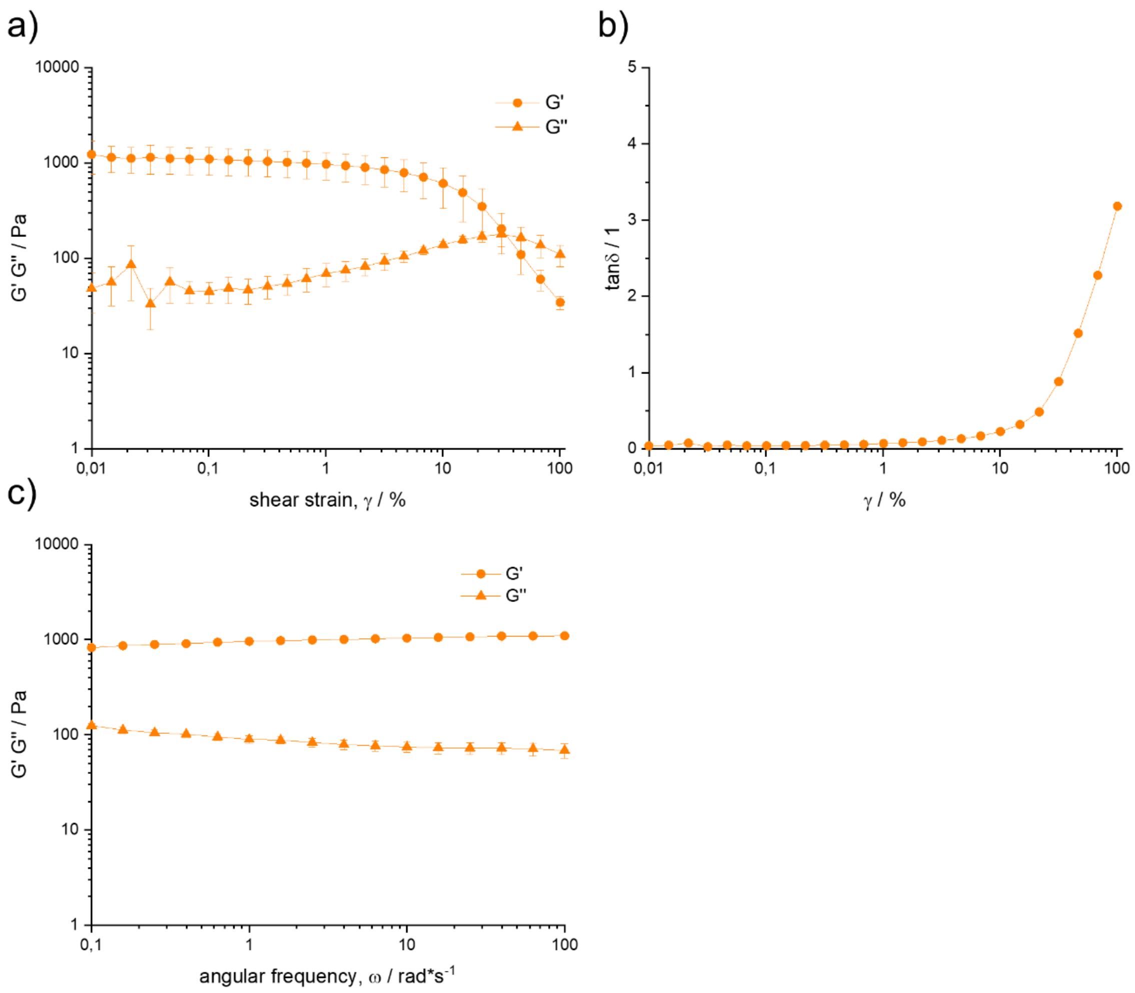

Figure 5. Rheological measurements of **DBD**(9.9), a) amplitude scan, b) loss tangent, c) frequency scan

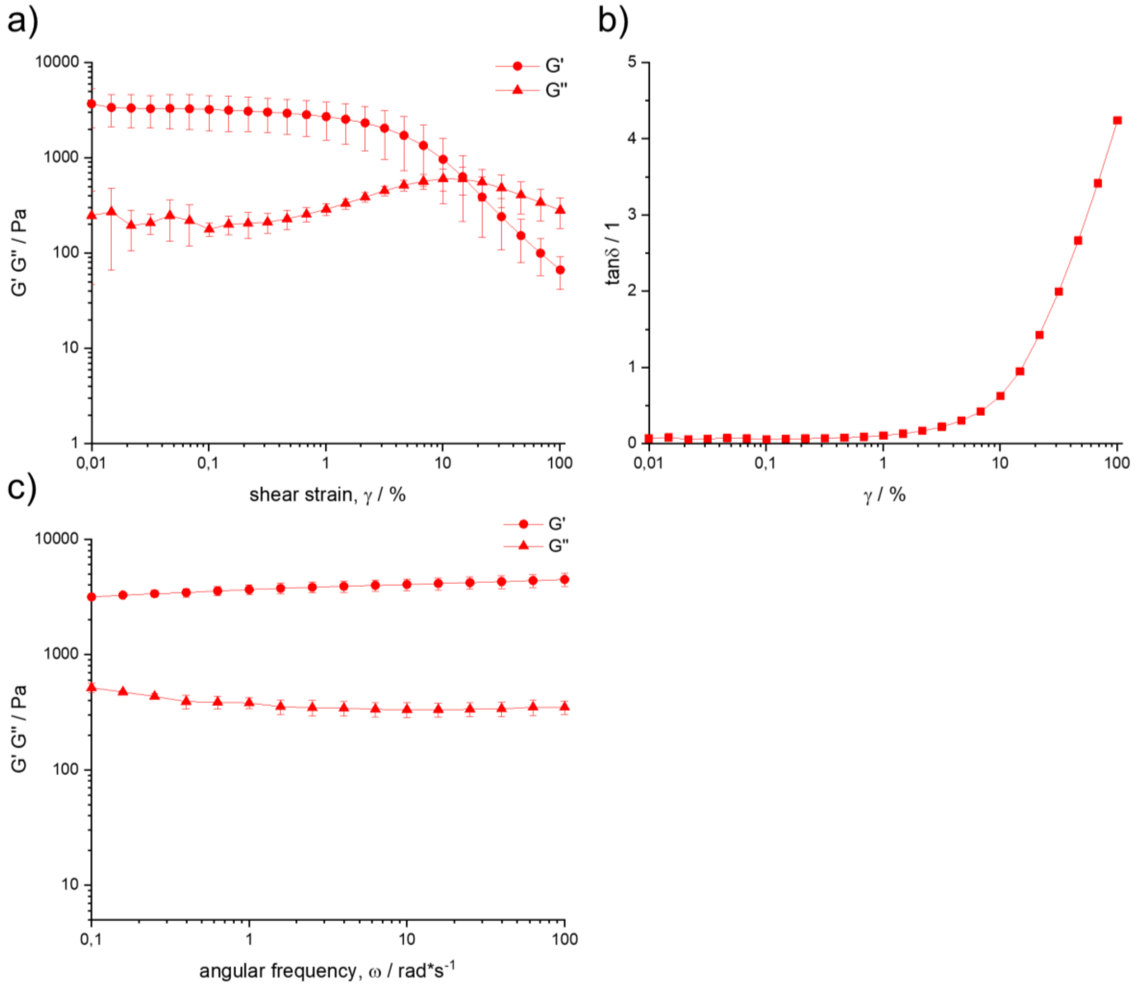

Figure 6. Rheological measurements of **DBD**(16.6), a) amplitude scan, b) loss tangent, c) frequency scan

7. Cyclic irradiation of **DBD**(0.7) in alkaline buffer

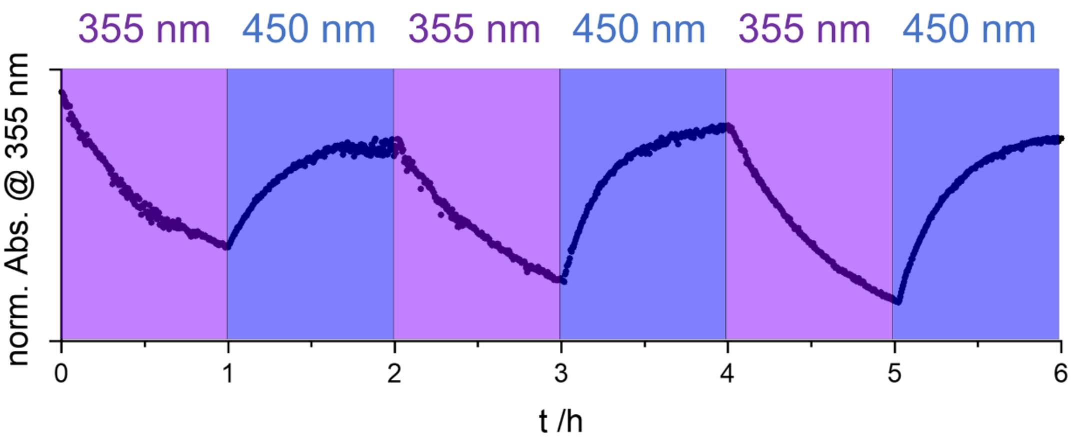

Figure 7. Cyclic irradiation of **DBD**(0.7) at 355 nm followed by 450 nm. Solvent is a pH 9.2 borate buffer.

8. Photoresponse of **DBD**(16.6)

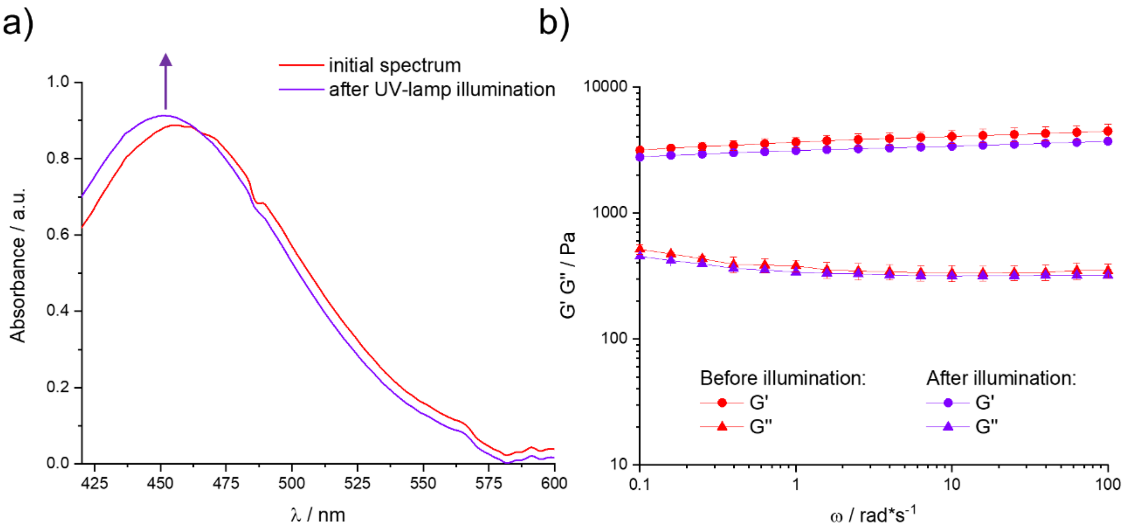

Figure 8. Figure a) shows the UV/VIS spectrum before (red) and after (violet) illumination with a UV lamp for 30 minutes. Figure b) shows photo-dependent frequency scans of the **DBD**(16.6) gel before (red) and after (violet) illumination.
